# Well-Informed and Willing, but Breastfeeding Does Not Work: A Qualitative Study on Perceived Support from Health Professionals among German Mothers with Breastfeeding Problems

**DOI:** 10.3390/healthcare10061009

**Published:** 2022-05-30

**Authors:** Mariz Spannhake, Charlotte Jansen, Tatiana Görig, Katharina Diehl

**Affiliations:** 1 Mannheim Institute of Public Health, Social and Preventive Medicine, Medical Faculty Mannheim, Heidelberg University, 68167 Mannheim, Germany; mariz_spannhake@gmx.de (M.S.); charlotte.jansen@medma.uni-heidelberg.de (C.J.); 2 Department of Medical Informatics, Biometry and Epidemiology, Friedrich-Alexander-Universität Erlangen-Nürnberg (FAU), 91054 Erlangen, Germany; tatiana.goerig@fau.de

**Keywords:** breastfeeding, breastfeeding problems, intention, support from health professionals, midwife, qualitative study, Germany

## Abstract

Although exclusive breastfeeding is recommended for the first six months of life, a large number of women worldwide do not practice it successfully. Our study aimed to investigate the perceived support from health professionals for women who experienced difficulties in breastfeeding. Furthermore, we examined women’s knowledge about breastfeeding and motivation to breastfeed. We conducted a qualitative exploratory study (June to October 2019) among 15 women who had experienced breastfeeding problems in Germany. Semi-structured interviews were audiotaped, transcribed verbatim, and analyzed using qualitative content analysis following Mayring’s approach. Prior to giving birth, the women’s motivation to breastfeed and theoretical knowledge were high, and breastfeeding problems were not anticipated. Difficulties in breastfeeding after giving birth created a highly demanding situation for the mothers. Health professionals were either perceived as very supportive, for example, by providing helpful technical advice or being emotional assistance, or they could be perceived as nonhelpful, thereby worsening the situation, for example, by pressuring the women to breastfeed or making improper remarks. Adequate support for young mothers in childbed consists of the provision of useful and realistic information concerning breastfeeding and a sensitive treatment when breastfeeding problems occur. Paying attention to this specific group of women who are experiencing difficulties in breastfeeding may improve maternal and child well-being and potentially enable breastfeeding.

## 1. Introduction

Breastfeeding problems are a common phenomenon in mothers and children [1,2]. Up to 70% of women experience difficulties that can range from a perceived lack of breast milk to cracked nipples or fatigue [3]. Previous studies found that these problems are one of the main reasons why women stop breastfeeding before reaching the recommended duration of exclusive breastfeeding by the World Health Organization (WHO) [3,4,5]. This situation is the same in different countries worldwide, including Germany, where many women wean too early due to breastfeeding problems [6,7]. Brettschneider et al. [6] found that among German mothers who do not meet the recommendations of the WHO, almost 70% stopped due to insufficient expression of breast milk. Since breast milk is undoubtedly the best nourishment for newborns, with positive health outcomes in the short and long run for both child and mother [8,9,10,11,12,13,14,15,16,17,18,19,20], it seems important to focus on these problems, eliminate them, and reach higher breastfeeding rates.

One aspect that may help reach a higher breastfeeding rate is support from health professionals. According to Leeming et al. [21], qualitative research may detect potential starting points to improve existing breastfeeding support. However, only a few studies provide findings on perinatal support from health professionals. For instance, the young parents in the study by Hoddinott et al. [22] were displeased with the mismatch between how easy breastfeeding was antenatally portrayed and idealized by health professionals and how difficult and problem-ridden it was in reality. In the study by Lee and Furedi [23], including women who had breastfeeding problems and those who decided not to breastfeed, information on bottle-feeding was withheld from women by health professionals. When they tried informing themselves, they realized how educational material on formula was limited, which was in sharp contrast to a large amount of information on breastfeeding [23]. Such experiences in a period that may be perceived as difficult due to the—often unexpected—breastfeeding problems may even worsen the situation. Trustful support from health professionals may be helpful in dealing with this situation.

Previous studies have shown that knowledge about breastfeeding and pre-birth intention to breastfeed is positively associated with the onset and duration of breastfeeding [24,25,26,27,28,29]. What remains unclear is whether women who face breastfeeding problems have sufficient information on and practical preparation for breastfeeding immediately after and even before birth. Therefore, we aimed to explore how informed and prepared women with breastfeeding problems are, that is, those who involuntarily had to stop natural breastfeeding early or were not able to breastfeed at all. In addition, we used our qualitative approach to describe perceived support from health professionals in the hospital immediately after giving birth and during aftercare by the midwife. Shedding light on these aspects may help to detect potential starting points for supporting women with breastfeeding problems in the short term and help increase breastfeeding rates in the long term.

## 2. Materials and Methods

We conducted a qualitative study among women who had problems with breastfeeding [30]. We used a convenient sample that was obtained via snowballing, midwives, and private contacts throughout Germany. Women were approached personally, face-to-face, via telephone, text message, or email—depending on the contact. We included women who had difficulties with breastfeeding or could not breastfeed at least one of their children and sufficient German language skills to participate in the interview. No further inclusion or exclusion criteria were applied. None of the contacted persons refused participation or dropped out.

Regarding the sample size, theoretical saturation was attained. We reached a point of saturation around the 12th interview, and this indicated that additional interviews would not lead to an increase regarding the gain of information being obtained. Therefore, we stopped recruiting participants after the 15th interview. We used semi-structured interviews with open-ended questions that were conducted either face-to-face or via telephone. This manuscript includes an analysis of the following main themes and their subthemes: intention to breastfeed, state of knowledge regarding breastfeeding in advance, and support from health professionals regarding breastfeeding and breastfeeding problems.

The interviews were conducted between 6 June and 22 October 2019 by the first author (M.S., female medical student, and graduate nurse) [30]. Prior to the interviews, M.S. was extensively trained by the last author (K.D.), who is an experienced interviewer. Participants were informed that M.S. has chosen this topic for her medical thesis and that her goal is to explore the situation of mothers who are unable to breastfeed or have to stop breastfeeding early. She went into the interviews with an open mind and was prepared for the fact that the women might become very emotional in the course of the interview.

The interviews lasted between 27 and 90 min (mean, 52 min). For the interviews, participants suggested locations where they felt comfortable talking and could not be disturbed. The same was suggested for interviews conducted via telephone. No third parties—except in some cases, the newborn or infant—were present during the interviews. All interviews were audiotaped and transcribed verbatim. Prior to the interviews, the participants were informed about the study’s procedure and data protection. All participants provided either written (face-to-face interviews) or oral (telephone interviews) consent to participate in the study. After the interview, all participants were provided with a €20 gift voucher as reimbursement for their time. Approval was obtained from the Ethics Committee II of the Medical Faculty Mannheim, Heidelberg University, on 26 March 2019 (number 2019-640N).

To analyze the 15 transcripts, we used qualitative content analysis following Mayring [31]. After having transcribed interviews verbatim, we thoroughly read the transcripts. The basis for the coding process was a set of main codes developed a priori based on the semi-structured interview guide. During the coding process, the code set was further refined and complemented [30]. We coded the data using the program MAXQDA (VERBI GmbH, Berlin, Germany, Version 20.3.0). The interviews were independently coded by two authors (M.S. and C.J.). Disagreements were discussed and resolved by consensus in each case. We systemized the transcripts by identifying common themes within the interviews by scanning for words and phrases used by the participants. Afterward, we summarized the findings descriptively.

Since qualitative research is not able to apply statistical methods to establish validity and reliability [32] of the research findings, we tried to enhance credibility of our research by adopting the following strategies: (1) We tried to describe our procedure, the characteristics of the interviewer as well as the characteristics of the participants as clear and as detailed as possible. (2) We acknowledge that we included a convenient sample. (3) We used a semi-structured interview guide that allows us to compare between interviews. (4) The interviews were coded independently by two coders, and codings were compared and discussed afterward. (5) We followed the concept of theoretical saturation. (6) We included verbatims of the participants in the results section to support our findings.

## 3. Results

### 3.1. Description of the Sample

Fifteen women who had problems with breastfeeding or could not breastfeed participated in this study. All women had problems with breastfeeding their first child. Two mothers had breastfeeding problems with the second child, too (M[other]05, M06), while two mothers were able to breastfeed their second child (M03, M14). Women were interviewed on average 33 months after breastfeeding problems occurred for the first time (Min: 6, Max: 66 months).

The presumed main reasons for these problems included the baby itself, early start of (additional) bottle-feeding, an insufficient amount of breast milk, and the mental condition of the mother. Further reasons were problems with correct latching, delivery through cesarean section, separation after birth, painful breast, potential genetic reasons, and health problems of the mother. The mean age (SD) of the mothers was 32.6 (5.3) years. The majority of them had one child (73%), three women had two children (20%), and one woman had three children (7%). When the participants gave birth to their first child, their mean age (SD) was 29.7 (5.3) years. The detailed demographic characteristics are shown in Table 1.

### 3.2. Before the Utilization of Breastfeeding Support

#### 3.2.1. Intention to Breastfeed

The intention to breastfeed mostly occurred at no specific point in time. To most of them, it “*has always been clear*” (M13) that they wanted to breastfeed (M01, M02, M03, M05, M09, M10, M11, M13, M14, M15). The others (M04, M06, M07, M08, M12) told the interviewer that their wish to breastfeed had emerged during the early stage of their pregnancy. They had not questioned their decisions at all. It was a general opinion to take breastfeeding for granted. Reasons for wanting to breastfeed were as follows: positive effects on the health of the child, more convenience than bottle-feeding, lower costs, positive effects on mother-infant attachment, positive effects on the health of the mother, and meeting social norms (Table 2). When initiating breastfeeding had not been considered previously, the decision was to at least try it.

#### 3.2.2. State of Knowledge

Women had a general knowledge of the positive health outcomes of breastfeeding for children and mothers (M01, M02, M03, M05, M06, M07, M08, M09, M10, M11, M12, M13, M14, M15). However, M04 said, “*I honestly didn’t know anything about it [i.e., breastfeeding]*”. and, therefore, had started to read specialist literature. One woman explicitly referred to the recommended duration of exclusive breastfeeding by the WHO (M01), and one woman mentioned the influence of maternal diet on the composition of breast milk (M10). Regarding practical skills, only one woman (M11) said that she had already known how to latch the newborn correctly, and M05 told the interviewer that she had already gathered a lot of experience with the children in her family. Others (M13, M14) tried to learn about breastfeeding positions by reading guidebooks.

Many participants stated, however, that they spent only limited time learning about breastfeeding in particular. They had believed that breastfeeding would come to them naturally and they would “*learn by doing*” (M14). “*To be honest, I didn’t think about it at all beforehand, and that is exactly how I tick. I believe it is a stepwise procedure. First, the child has to be here, and then we will see how it works*”. (M10). In retrospect, M14 recognized that she “*could not imagine that it [i.e., breastfeeding] would be so difficult*”. Others had been surprised that breastfeeding was not easy at all for them. M07 noted, “*Maybe I should have done a little more research [on breastfeeding]*” and:

“*I always believed it works automatically. At first, I actually did not know what I was going to do with the child (laughs): where and how to/not to position the child in order to breastfeed. Somehow, one is more unprepared than one thinks*”.

(M09)

### 3.3. Support from Health Professionals

#### 3.3.1. Information and Support from Health Professionals before Birth

Attending a birth preparation course was popular among the participants (M01, M02, M03, M04, M06, M08, M09, M12, M14, M15). While most of the women had received, at least to some extent, information on breastfeeding in these courses, others did not obtain as much information as they desired or any preparation at all. M12 had not been able to attend the course to the end due to preterm birth, and M03 stated that “*Retrospectively, I must say that I am quite astonished because it really is a very large part of a mother’s first tasks to breastfeed a child; the birth preparation course did not play a role in this*”. Some women had mentioned that health professionals ignored them when they asked for further information. “*They just said, ‘Don’t worry about that. It will all work!’*” (M04). Undergoing practical exercises on how to breastfeed with a doll during the course was rare (M01, M08).

M01 clearly indicated that information on formula-feeding was limited, and a few participants (M03, M09) conceded that later on when they experienced breastfeeding problems, they were overwhelmed with bottle-feeding their children. M09 retrospectively also wondered why breastfeeding problems had not been addressed in the birth preparation course. “*During the birth preparation course, they always said, ‘Yes, there can be breastfeeding problems.’ However, the details of the problem were not clear to me*”. (M09).

#### 3.3.2. Support from Health Professionals after Birth

After giving birth, participants reported being technically advised concerning breastfeeding by health professionals during their hospital stay (M01, M03, M04, M05, M06, M08, M09, M10, M11, M12, M13, M14, M15) and being supplied with useful materials, such as nipple shields, nipple ointment, or a breast pump (M01, M02, M03, M04, M05, M06, M08, M09, M10, M12, M14, M15). Gratefulness for the helpful counseling was occasionally expressed during the interviews: “*So the midwives and nurses I had in my ward provided help regarding breastfeeding; I would go there repeatedly. They provided the best support and advice and they were there for me*”. (M11), and “*The nurse was just wonderful. She tried everything and provided support with breastfeeding*”. (M10).

However, some participants reported asking for help to no avail. “*They were all super friendly, and they said if I needed help, I should let them know. The implementation was not so great, because everyone had a lot to do, of course*”. (M07). M08 was completely disappointed with the overall care in the hospital and said that it was “*[…] bad. Very, very bad*”.

Some of the women were irritated by health professionals who had different opinions regarding breastfeeding (M01, M03, M07), e.g.,:

“*What made me insecure right from the start was, um, that, at the hospital, […] about three pediatric nurses came into the room during the day and each one somehow had a different opinion from the others on how this [i.e., breastfeeding] should go*”.

(M03)

M07 stated that “*[…] they were also of divided opinions [i.e., concerning breastfeeding]. One said, ‘At least every four hours’, the other, ‘No, let him sleep’, the other, ‘Just not at night!’, and the other, ‘Yes, definitely at night!’*”.

#### 3.3.3. Support from Health Professionals When Experiencing Breastfeeding Problems

When experiencing breastfeeding problems, several women felt as if they were not taken seriously and found the health professionals to be insensitive (M01, M02, M03, M04, M05, M13, M14, M15). M13 said, “*And when we were at home, the midwife simply said to me, ‘breastfeeding often doesn’t work after a cesarean birth’ and didn’t really help to try anyway*”. When M02 tried to at least partly meet her breastfeeding goals by pumping breast milk for her firstborn, who had a cleft lip and palate, she did not find the physician supportive. “*The oral and maxillofacial surgeon more than belittled my effort to pump breast milk*”. (M02). Moreover, mothers felt pressured (M01, M03, M05, M07, M13, M14, M15). For instance, M07 subsumed, “*I was exposed to a lot of pressure, from the pediatrician among others*”. This even led to misinforming women to encourage them to breastfeed at any cost (M07, M15). “*She [i.e., the nurse] then said, ‘Yes, when you pump, the milk goes back.’* *That is not true at all, as breast milk is stimulated*”. (M15). Very negative and devaluating situations with health professionals were experienced when breastfeeding did not work, such as being confronted by strong breastfeeding supporters (M04, M05, M15). “*When I was asked if I was still breastfeeding and I said no, the only reply from the pediatrician was ‘What are you doing to your child? Breastfeeding is the best thing that you can do.’*” (M05). M15 experienced one particular situation in the hospital in which she was completely exhausted from breastfeeding and in which the midwife refused to provide her the amount of formula as requested but imputed her a lack of desire to breastfeed. “*And then she [i.e., the midwife] looks at me and says, ‘If you don’t want to breastfeed, then stop.’ You felt so bad that you decided you don’t want to breastfeed anymore, because you cannot. That was so reproachful*”. (M15).

Other women said that they did not have the impression that not being able to breastfeed changed anything in the treatment they received from hospital staff (M05, M09, M12). However, participants also emphasized the emotional support they received from health professionals (M02, M03, M04, M05, M07, M08, M09, M10, M11, M12, M13, M14, M15), especially from their midwives at home (M02, M03, M04, M05, M08, M09, M10, M12, M15). M08 summarized:

“*However, luckily, I had a really great midwife who said, ‘Come on, relax and do not stress yourself. Do not focus so much on the fact that he is drinking now, but focus on the fact that you get a feeling of what it is like to deliver milk, first*.’”

(M08)

Midwives were often seen as the most helpful conversational partners when talking about the difficult breastfeeding situation (M02, M03, M06, M08, M12, M13, M14), whereas none declared a physician or a specialized breastfeeding advisor as an important dialog partner. Furthermore, M09 found a nurse working with an aid agency to be the most helpful assistant.

### 3.4. Evaluation of the Support from Health Professionals

Altogether, the entirety of health professionals was considered to be strong breastfeeding supporters (M03, M05, M13, M14). However, others had the impression that health professionals saw breastfeeding as the best option but also knew that in some cases, formula-feeding was justified (M02, M09, M10, M11, M12). Yet, however, others reported experiencing both very supporting and differentiated views from health professionals concerning breastfeeding (M01, M07, M15). M01 and M07 mentioned that “*the midwives or the nurses did not agree with one another*” (M01). M15 mentioned that there was a difference between the hospital staff and her aftercare midwife, in which the latter was “*completely relaxed [regarding the breastfeeding topic]*” (M15). However, M04 told the interviewer that her midwife favored formula-feeding, and two women stated that the health professionals in the hospital were rather neutral regarding how to feed the newborn (M06, M08).

### 3.5. Desired Support from Health Professionals for Future Pregnancies

Many of the participants wished that the health professionals would provide more help and advice to them. This was also related to the women’s wish for the health professionals to create more time for their needs. Several women also mentioned the lack of competent advice as something to improve on. More sensitive treatment and, in particular, the reduction of pressure were desired changes for the better. However, some also emphasized that they were satisfied with the overall care and actually had no real suggestions for improvement (Table 3).

## 4. Discussion

Our study showed that the participants wished to breastfeed and were mainly motivated by the potential positive health effects for the child. The women were well-informed, as many of them attended a birth preparation course, where they gathered theoretical knowledge; however, practical knowledge was low. Based on this, most participants perceived breastfeeding as being natural and were surprised when they were confronted with problems.

After giving birth, nearly all women received technical advice on breastfeeding from health professionals and experienced emotional support when breastfeeding did not work, especially from their aftercare midwives. However, some participants also experienced negative situations with health professionals and perceived, for instance, insensitive, pressuring, and devaluative treatments. In many cases, the women felt as if their needs and questions were being ignored at the hospital. Some reported that different health professionals had different opinions on breastfeeding, and this irritated the women. Overall, the participants in this study would wish for more attention to be paid to them and advice, in addition to the consensus and empathy from health professionals in potential future pregnancies.

### 4.1. Comparison with Previous Research

In line with our findings, previous studies have shown that the intention to breastfeed is often developed before or during the early stage of pregnancy [25,33]. Many studies have revealed that the motivation to initiate breastfeeding is high [25,28,34,35,36,37], and that this motivation is associated with the onset and duration of breastfeeding after childbirth [25,26,27,28,29]. In our study, we focused on women who were faced with breastfeeding problems and therefore had to stop breastfeeding early, although we found that motivation was high, and all participants had the intention to breastfeed.

In line with another study, the most frequent reasons for intending to breastfeed were the benefits for the infant’s health, whereas the benefits for the mother herself did not play such an important role [33]. This shows that the participants had dealt with this topic before, at least theoretically. However, as described in previous studies, the idea that breastfeeding would be natural and without difficulty was widespread among our participants [38,39,40,41,42,43]. Thus, when breastfeeding problems occurred, women were surprised and overwhelmed by the unexpected situation. In addition, women in our study and those in previous studies reported that in some cases, health professionals during the birth preparation course were unwilling or reluctant to inform them about formulas and bottle-feeding [23,39,43,44,45]. This may lead to even more pressure for the women apart from what they are already faced with: they are willing to breastfeed, they know about the positive health effects for the child, but they are unable to breastfeed. This situation is very sensitive, and these women require a lot of attention from health professionals. They need support (e.g., advice regarding the use of nipple shields because they may succeed in breastfeeding in the long run), education (e.g., on the use of breast pumps to provide them the feeling that they can, at least, provide the newborn breast milk), and alternatives (e.g., information on formula when they do not succeed with nipple shields or the breast pump). Based on this balanced approach, an individualized solution can be chosen to provide the newborn with the best possible nourishment while also focusing on the mental well-being of the mother.

We found that midwives play an important role in the overall care of young mothers in this very vulnerable phase in a woman’s life. In a previous study, two main types of midwives were identified: those who are rather “breast centered” and mainly provide technical advice to women on how to feed the child and those who are rather “women centered” that accompany young mothers and have a greater focus on the woman’s well-being [46]. However, in our study, many participants reported that their aftercare midwives were very helpful and assisting breastfeeding experts, who focused not only on technical breastfeeding education but also provided emotional support. This difference from the above-mentioned study results might be explained due to the fact that the home care midwives were underrepresented in the review study by Swerts et al. [46]. Successful breastfeeding support may, therefore, not solely include technical advice but also involve respecting the woman’s individual needs.

Our participants’ experiences with various health professionals when they perceived breastfeeding problems ranged from very positive to quite negative. In line with this, previous research showed that, while some health professionals were very sensitive toward women in childbed and were, therefore, quite helpful [23,40,43], others acted rather “bossy” [43], cruel [23], or verbally pressured the women to continue breastfeeding [22,23,38,40]. Other studies reported that women simply felt judged when formula-feeding [44,47]. Regarding this aspect, participants in our study wished that the health professionals would be more empathic and understanding, although they were aware that breastfeeding is the best choice. Previous studies have shown that a relaxed environment and an unstressed and self-confident woman who is treated gently and receives the support she needs are important factors for successful breastfeeding [38,48].

Inconsistent and different information on how to breastfeed, as some of the participants in our study mentioned, was also reported in other studies [38,40,45,49]. In order to prevent confusion, it would be helpful to decide on a specific way of providing breastfeeding support to each woman in childbed by health professionals and to stick to it until a new strategy is discussed. This also includes the aspect of time spent with women, as previous studies found that a lack of time makes it difficult to practice breastfeeding with the newborn [38,46,48].

Based on our findings and the findings from previous studies, different ideas for future research can be developed. In our study, different aspects were mentioned in the interviews that we were not able to investigate in detail, for instance, the variety and nature of different breastfeeding problems. Moreover, further research may focus on the effect of different care models for women in childbed and which impact a home care system with one responsible midwife can have on breastfeeding compared to other care models.

### 4.2. Limitations

First, as our qualitative study consisted of a convenience sample extended by snowballing, the generalizability of our findings may be limited [30]. However, generalizability is not the main aim of qualitative research. Instead, we explored the perinatal situation of women who had breastfeeding problems to gain in-depth insights into perceived breastfeeding support from health professionals. Second, we cannot ignore the possibility that the participants were unable to correctly recall each situation they talked about and that reports on encounters with health professionals recalled might have been distorted due to their emotions. Third, women who experienced very difficult situations in health care might have been more willing to participate in this study just to share their negative experiences. However, these interviews helped to identify potential starting points for supporting this specific group of women, who are well-informed and intend to breastfeed, but encounter problems. Therefore, our study provides valuable insights into the perinatal situation of women who face breastfeeding problems and seek help from health professionals.

### 4.3. Implications for Practice

Our study provides important suggestions for handling women with breastfeeding problems. Providing balanced and realistic education concerning breastfeeding, potential problems, and bottle-feeding as an alternative during pregnancy may help young mothers to anticipate difficult situations when it comes to breastfeeding. Spending enough time with the women and providing them with support (e.g., providing nipple shields) without pressure may help them overcome problems and successfully breastfeed. It might also be helpful to address potential breastfeeding problems and the alternative of formula-feeding during birth preparation courses, which was not thematized in the courses attended by our participants. Mothers who know the details about specific problems that can occur during the first time with a newborn may be able to better cope with potential problems. Since mothers’ intention to breastfeed is high, education on potential problems and alternative forms of feeding will probably not weaken this motivation. However, informing all women about formula-feeding in the birth preparation course may help those mothers who have to formula-feed to feel less stigmatized [30].

Midwives in the aftercare have great influence and should be aware of their impact. They are an important person of trust and, therefore, a helpful player in the situation when breastfeeding problems occur. Home care midwives are able to provide advice that suits the individual situation.

It is important to provide a sensitive way to deal with this specific group of women who aim to breastfeed and are informed about the positive health outcomes for the child but are totally devastated due to the inability to breastfeed. Providing them the feeling of being a good mother, whether they breastfeed or not, might help them to overcome this situation. Informing these women that breastfeeding problems are quite common and that it might not always work out as easy as believed may help them to cope with potential feelings of guilt and self-blame.

## 5. Conclusions

We found that women can be faced with breastfeeding problems, although they intended to breastfeed, are highly motivated by the potential positive health outcomes for the child, and are well-informed. These women require specific and sensitive care in hospitals and during aftercare. Additionally, they need information, support, and time to overcome the experienced disappointment and, possibly, be able to breastfeed in the long run. Therefore, inconsistent opinions and pressure from health professionals may be counterproductive. Particularly, midwives play an important role, as they act as trusted persons.

Health professionals and especially midwives in aftercare can be a valuable help for mothers experiencing breastfeeding problems. Their support can be the key to improving maternal well-being and potentially enabling breastfeeding in the end.

## Figures and Tables

**Table 1:** Demographic Characteristics of the Participating Women Who Reported Problems in Breastfeeding for at Least One of Their Children (*n* = 15).

ID	Age of Woman, Years	Number of Children with Breastfeeding Problems	Age of Firstborn Child, Months	Age of Second-Born Child, Months	Age of Woman at First Child’s Birth, Years	Highest Vocational Qualification	Relationship at Time of First Child’s Birth
M01	34	1	25		31	doctorate	Yes
M02	32	1	48	22	28	completed vocational training	Yes
M03	37	1	37		34	university degree	Yes
M04	28	1	28		26	university degree	Yes
M05	25	2	76	25	18	none	No
M06	34	3	39	12 ^a^	31	completed vocational training	Yes
M07	22	1	9		21	completed vocational training	Yes
M08	29	1	16		27	completed vocational training	Yes
M09	35	1	29		33	university degree	Yes
M10	36	1	42		33	completed vocational training	Yes
M11	28	1	25		26	completed vocational training	Yes
M12	41	1	66		35	completed vocational training	No
M13	33	1	14		32	university degree	Yes
M14	40	1	33	11	37	university degree	Yes
M15	35	1	6		34	completed vocational training	Yes

^a^ twins.

**Table 2:** Reported Reasons for Intending to Breastfeed.

Subtheme	Reported by	Examples for Quotes
Positive effects on the health of the child	M01	I: “And why did you decide, um, to breastfeed at all?”
	M02	M04: “Well, you try to give the child the best you can”.
	M04	M09: “That was not really a question for me, so it [i.e., breastfeeding] was the most natural thing for me, uh, and since you also know that breast milk is actually the best for the child, uh, I really wanted to do that”.
	M05	
	M06	
	M07	
	M08	M11: “I think it [i.e., breastfeeding] is just beautiful and also important for the child, because you simply transfer the immune system, that you have yourself, to the child […]”
	M09	
	M10	
	M11	M12: “Um, I myself am an allergy child. And they say that breast milk […] can also protect against allergies and that was just important to me”.
	M12	
	M13	
	M14	I: “And why did you even want to breastfeed back then?”
	M15	M15: “Because you thought it was the healthiest thing for the child”.
Breastfeeding is taken for granted	M01	M01: “So for me it was always clear that breastfeeding is part of it”.
	M02	M08: “Actually this is (..)/it was clear to me. I did not have to think back and forth whether to breastfeed or not. It is the healthiest and best thing for the child and that is why it was clear to me, ‘I am going to breastfeed!’”
	M03	
	M04	
	M05	
	M06	I: “And why did you even want to breastfeed?”
	M07	M10: “[…] That was part of the natural birth for me. There were two things I really wanted: to give birth the natural way and breastfeed once. And why? Um (.) because, firstly, I think it is the most natural thing, because since mankind has been around/nature built it in so that it works, so it makes sense, too. […]”
	M08	
	M09	
	M10	
	M11	
	M13	
More convenience than with bottle-feeding	M06	M06: “It is simply breast milk. It is simply practical, you can go anywhere and do not have to carry anything with you, all the bottles and so on”.
	M09	
	M14	
	M15	M14: “[…] because there is nothing easier than, ‘Breast out, child on’. You always have the same temperature (laughs) and of course it is also a comfortable aspect that you always have it with you and do not have to pack bottles […]”
Lower costs	M02	M13: “Yes, you always heard, ‘This is the best for the child!’ And you want to give the best for the child, instead of spending money on some milk powder, (smiles) which was also a fact”.
	M06	
	M13	
Positive effects on mother-infant attachment	M07	M07: “[…] it [i.e., breastfeeding] (.) is also for the mother-infant attachment, uh, also a great building block, of course, that also contributes to it […]”
	M11	
	M12	
Positive effects on the health of the mother	M02	M02: “I never actually considered buying powdered milk in any way. It was always clear to me that I wanted to breastfeed, because it is simply the best and is also good for my health […]”
	M06	
Meeting social norms	M07	M07: “Well, by and large it came from me. Of course, there is also social pressure behind it, because everyone says, ‘Yes, you will definitely breastfeed?’ […]”

**Table 3:** Desired Future Breastfeeding Support from Health Professionals.

Subtheme	Reported by	Examples for Quotes
To provide more help and advice	M02	M04: “Um, and then the midwife reluctantly wanted to show it [i.e., breastfeeding] to me. So she grabbed the breast and somehow tried to do that/but [name of the child] was already screaming, so that he could not do it anymore/that he did not understand that he should drink now. (.) But that was the only help”.
	M04	
	M05	
	M07	
	M08	M05: “I could not put her [i.e., the newborn] on and nobody helped me”.
	M09	M13: “[…] I think that’s why they [i.e., the health professionals] try that, too [to support breastfeeding]. In principle, it is not wrong, but well, they would have to really help you with that. Maybe show latching positions […]”
	M10	
	M12	
	M13	
	M14	
To provide more competent advice	M02	M02: “Yes, that the midwives and also […] the nursing staff on the wards simply are a lot more trained and somehow have more idea on it [i.e., breastfeeding] and can tell you what other possibilities there would have been. For example, tell about this breast feeding set or that you get properly shown how you actually pump instead of placing the breast pump there and leaving you all alone with it. ‘Yes, then do it and have a nice day.’ […]”
	M03	
	M07	
	M08	
	M10	
	M12	
	M13	M08: “But unfortunately I have to honestly admit that the hospital has already screwed it up. I think/I am pretty sure that if I had been properly shown in the hospital how, how that/how to see that the child is actually drinking, then it would not have come to that. (clears her throat)”
	M15	M10: “Well, I do believe that they [i.e., the midwives] like to show how it [i.e., breastfeeding] is done and that is what they did. But I think as soon as someone really has problems, from that point on—and that was with me—that is where it ends. I would guess. Would there have to be someone else at the hospital who knows his way around a bit better? […]”
To create more time for the mothers’ needs	M04	M07: “I think the biggest problem was just that I could never put him on properly. Because they [i.e., the midwives] just did not have enough time to show me properly”.
	M07	
	M08	
	M09	M12: “[…] But as I said […] I had already given birth […] I missed it so much that it [i.e., the preliminary meeting with the midwife] had not been done! […]”
	M12	
	M13	M14: “So we were just a lot of women who had recently given birth in the hospital and then they were understaffed, the midwives. And yes […] they just put the baby on, head on, bang, and with such a speed that you actually could not see it properly, how does it [i.e., latching] work now?”
	M14	
To be more sensitive	M01	I: “What would you have wished for from the midwives?”
	M05	M05: “(.) Maybe a little more understanding. Maybe a little more sensitivity”.
	M06	M09: “[…] the way they [i.e., the health professionals] did it [i.e., the way they helped] was not always the friendliest […]”
	M07	
	M09	
	M14	
To pressure less	M01	M01: “[…] Um, if the other [i.e., the midwife] had also been understanding, I think that would have helped me a lot. And not this doggedness, ‘You have to give it your all now!’ Because I was really/I was devastated. I could not do it. But (.) that has not been accepted as an answer (breathes in). […]”
	M14	
	M15	
Altogether satisfied with the overall care from health professionals	M03	M03: “[…] but in general I have to say […] I also very, very willingly received support there, yes”.
	M06	
	M11	I: “Is there anything else you would have wished for?”
		M06: “Nothing, you know what it is about and if I had not known something, I would have asked”.

## Data Availability

Data are available from the corresponding author upon reasonable request.
